# Fiber optic Raman spectroscopy for the evaluation of disease state in Duchenne muscular dystrophy: An assessment using the *mdx* model and human muscle

**DOI:** 10.1002/mus.27671

**Published:** 2022-07-15

**Authors:** James J. P. Alix, Maria Plesia, Sarah A. Hool, Ian Coldicott, Catherine A. Kendall, Pamela J. Shaw DBE, Richard J. Mead, John C. Day

**Affiliations:** ^1^ Sheffield Institute for Translational Neuroscience University of Sheffield Sheffield UK; ^2^ Neuroscience Institute University of Sheffield Sheffield UK; ^3^ Biophotonics Research Unit Gloucestershire Hospitals NHS Foundation Trust Gloucester UK; ^4^ Interface Analysis Centre, School of Physics University of Bristol Bristol UK

**Keywords:** biomarker, Duchenne muscular dystrophy, exercise, mdx mouse, muscle necrosis, Raman spectroscopy

## Abstract

**Introduction/Aims:**

Raman spectroscopy is an emerging technique for the evaluation of muscle disease. In this study we evaluate the ability of *in vivo* intramuscular Raman spectroscopy to detect the effects of voluntary running in the *mdx* model of Duchenne muscular dystrophy (DMD). We also compare *mdx* data with muscle spectra from human DMD patients.

**Methods:**

Thirty 90‐day‐old *mdx* mice were randomly allocated to an exercised group (48‐hour access to a running wheel) and an unexercised group (n = 15 per group). *In vivo* Raman spectra were collected from both gastrocnemius muscles and histopathological assessment subsequently performed. Raman data were analyzed using principal component analysis–fed linear discriminant analysis (PCA‐LDA). Exercised and unexercised *mdx* muscle spectra were compared with human DMD samples using cosine similarity.

**Results:**

Exercised mice ran an average of 6.5 km over 48 hours, which induced a significant increase in muscle necrosis (*P* = .03). PCA‐LDA scores were significantly different between the exercised and unexercised groups (*P* < .0001) and correlated significantly with distance run (*P* = .01). Raman spectra from exercised mice more closely resembled human spectra than those from unexercised mice.

**Discussion:**

Raman spectroscopy provides a readout of the biochemical alterations in muscle in both the *mdx* mouse and human DMD muscle.

**Abbreviations**
airPLS: adaptive, iteratively reweighted penalized least squares
DMD: Duchenne muscular dystrophy
LDA: linear discriminant analysis
LDF: linear discriminant function
PCA: principal component analysis
PC: principal component

## 1 INTRODUCTION

Translational biomarkers are required for the evaluation of disease across a range of neuromuscular disorders. Raman spectroscopy is an emerging method for the evaluation of neurological diseases,^1^ including muscle pathology.^2^ In this technique, light of a single wavelength is used to stimulate the vibrational modes of molecules in a sample. Most of this light is elastically (or “Rayleigh”) scattered; that is, it returns with the same energy. However, a small number of photons are inelastically scattered and have their energy changed, this is known as the “Raman effect.” The energy change depends on the molecules encountered in the sample, and thus, through collection of only the inelastically scattered light, Raman spectroscopy provides a molecular fingerprint of the tissue^3^ (Figure S1). It is rapid and label‐free, requiring no sample preparation. Biomedical applications of the technology are gaining momentum across many different areas,^4^ including neuromuscular diseases.^2^, ^5^, ^6^

Microscope formats are traditionally used to evaluate tissue or biofluid samples, but recent developments in fiber optic technology are of increasing interest.^7^ Such methods hold the promise of targeting light to an area of interest *in vivo* to provide a rapid assessment of tissue health. We have recently demonstrated the potential of this approach for the evaluation of muscle pathology, using a miniaturized probe to evaluate muscle health *in vivo* in preclinical models of both Duchenne muscular dystrophy (DMD) and amyotrophic lateral sclerosis.^5^

In the present study we have further assessed the utility of fiber optic Raman spectroscopy as a translational biomarker for DMD. The most widely studied animal model of DMD is the *mdx* mouse, which carries a null mutation of the dystrophin gene due to a premature stop codon and manifests a relatively mild phenotype.^8^ The model manifests an acute onset of muscle fiber necrosis at around 30 days of age, followed by chronic, low‐level pathology.^9^ This can make detection of interventions difficult to detect in adult mice and, as a result, exercise paradigms are often undertaken. Bursts of exercise, particularly high‐intensity exercise, can be used to induce muscle damage such that the pathology more closely resembles the human disease.^10^, ^11^ These effects are in contrast to those of chronic, lower intensity exercise regimes, which may have a protective effect in both the *mdx* model^12^, ^13^ and humans.^14^ Several different methods of exercise have been utilized across both acute and chronic regimes, including swimming, forced treadmill running, and voluntary wheel running^9^, ^15^; of these, voluntary wheel running is arguably the easiest and least invasive to implement.

In this study we tested the hypothesis that Raman spectroscopy of muscle will be able to detect and quantify the effect of the running wheel intervention. Because the changes induced by exercise are known to result in histopathological features more akin to the human disease, we also hypothesized that Raman spectra from exercised mice would more closely resemble those obtained from human DMD muscle samples.

## 2 METHODS

### 2.1 Animal model

The *mdx* C57/Bl10 model of DMD was used in this study. Mouse breeding was undertaken in a specified pathogen‐free environment, with transfer to a standard facility (12‐hour light/dark cycle and room temperature 21°C) for experimental work. All experimental procedures involving animals were undertaken with the approval of the University of Sheffield ethical review subcommittee and UK Home Office (License No. 70/8587), in accordance with the Animal (Scientific Procedures) Act of 1986. The ARRIVE guidelines^16^ were followed in the conduct of this work.

### 2.2 Running wheel paradigm

Female mice were used for the study. A total of 30 mice were randomly allocated to exercised and unexercised groups (n = 15 per group). The exercised mice were caged individually for a total of 48 hours with a 37.8‐cm circumference plastic Fast Trac running wheel (Bio‐Serv). The wheel was attached to a 4‐cm post fixed to the floor of the cage and placed in the corner of each cage.^17^ A magnet was attached to the underside of the wheel and a bicycle computer with a reed switch was attached to the side of the cage. Mice exercised voluntarily on the running wheel for 48 hours and the distance completed by each individual mouse was measured and recorded daily. The exercise duration was limited to 48 hours to prevent muscle regeneration after exercise‐induced necrosis complicating the background pathology.^10^ Food and water were administered ad libitum. After the *in vivo* Raman recordings mice were humanely euthanized and gastrocnemius muscles dissected for either *ex vivo* Raman recording or histology.

### 2.3 Raman spectroscopy

The *in vivo* fiber optic methodology was undertaken as previously described.^5^ Briefly, mice were anesthetized (2% isoflurane) and recording performed on a heat pad to aid thermoregulation. Hindlimbs were shaved and the fiber optic Raman probe inserted into the medial and lateral heads of gastrocnemius bilaterally (resulting in four spectra per mouse). The fiber optic Raman spectra were obtained using a Raman probe housed within a 21‐gauge hypodermic needle. The incident light was provided by an 830‐nm laser (power output 60 mW at the probe tip) and the probe optically paired to the spectrometer for efficiency (see Figure S2 for equipment schematic). The Raman signal was recorded by averaging 10 × 4‐second epochs, resulting in a total recording time of 40 seconds for each placement of the probe. After *in vivo* recordings, mice were humanely culled (cervical dislocation) and muscles dissected. These were then snap frozen in liquid nitrogen and stored at −80°C until required for further analyses. Five gastrocnemius muscles (from n = 5 mice) were taken for ex vivo Raman recordings, these were thawed to room temperature and the fiber optic probe inserted into the muscle. Spectra were collected using the same parameters noted earlier.

### 2.4 Histology

Gastrocnemius muscle samples from 13 mice (n = 7 exercised, n = 6 unexercised) were cryosectioned at 10 μm and mounted on uncoated, charged slides. To gain a representation of pathology throughout a reasonable portion of the muscle, sections were first cut to approximately one third of the way into the muscle. Twenty sections were then cut and collected (four per slide), after which a further 100 μm was discarded and then another 20 sections were taken. This was repeated four times. One slide at each depth was then randomly selected for further staining and analysis (resulting in analysis of 16 sections per mouse).

Hematoxylin‐and‐eosin staining was performed. Sections were first left at room temperature to thaw for 30 minutes and then placed in 95% alcohol for 5 minutes and then cleared in water. Sections were then stained in Harris hematoxylin for 2 minutes and washed in water. After being washed in Scott's water for 1 minute, the slides were stained in eosin for 5 minutes, washed in water again, and then dehydrated in alcohols (75%, 90%, 100%, 100%). The sections were then cleared in xylene and mounted in DPX. Slides were imaged using a digital slide scanner (Nanozoomer series; Hamamatsu Photonics, Tokyo, Japan). Whole cross‐sections were analyzed using ImageJ. Areas of necrosis were identified by infiltrating basophilic inflammatory cells and degenerating myofibers with a pale and often fragmented sarcoplasm.^18^, ^19^ The amount of necrosis was reported as a percentage of the muscle cross‐section.

### 2.5 Human samples

Quadriceps muscle samples from boys with genetically confirmed DMD (n = 3; ages 2, 4, and 10 years) were obtained from the Oxford Brain Bank and stored at −80°C. Use of the samples in research was approved by a local NHS research ethics committee (Yorkshire and Humber‐Sheffield, Reference No. 16/YH/0261). Raman spectra were obtained by gently pressing the fiber optic probe against the sample in five to seven visually discrete locations. Spectra were collected with the same recording parameters used in mice.

### 2.6 Statistical analysis

Analysis was done in MATLAB 2019a (The MathWorks) using custom scripts. Raman spectra were interpolated and windowed in the biological fingerprint region between 900 cm^−1^ and 1800 cm^−1^ to avoid silica background in the optical fibers (at <900 cm^−1^) and uninformative noise >1800 cm^−1^. Spectra were then smoothed (second‐order Savitzky‐Golay filter, 9‐datapoint window width) and spectral background was removed using the adaptive, iteratively reweighted penalized least‐squares (airPLS) algorithm.^20^ Data were then normalized (standard normal variate normalization). To identify and quantify exercise‐induced molecular changes in muscle, multivariate analyses were used. Data were mean centered and principal component analysis (PCA) was performed. PCA is an unsupervised technique that aims to simplify the data by finding a reduced number of variables (principal components, PCs) to explain the underlying variance, without information on the groups (ie, the algorithm does not know there are exercised and unexercised groups). *T‐*tests on the first 10 PCs were undertaken with a false‐discovery rate correction (*Q* = .01) to identify the PCs for the linear discriminant analysis (LDA), thus enabling PCA‐fed LDA, which is one of the most common data analysis techniques applied to Raman spectroscopy.^3^, ^21^ LDA is a supervised methodology, that is, it considers the groups. The algorithm finds directions (or linear discriminant functions) within the feature space (the PC space in this instance) that maximize the separation of the exercised and unexercised groups, while minimizing the intragroup variance. The linear discriminant function (LDF) scores represent a linear combination of the PCs obtained for each sample. The linear discriminant (LD) loadings plots represent the extent to which each individual wavenumber contributes to the LD and can thus be plotted like a spectrum to illustrate the important peaks in the LDA process. Nested *t*‐tests of linear discriminant function scores (spectra nested within mice) were performed using GraphPad Prism (version 9). As Raman spectra are complex, examining small spectral windows can give insight into the similarities and differences of specific features.^22^ Cosine similarity, which represents the cosine angle between two vectors,^23^ was thus assessed using spectral intervals of 100 cm^−1^.

## 3 RESULTS

**FIGURE 1:**
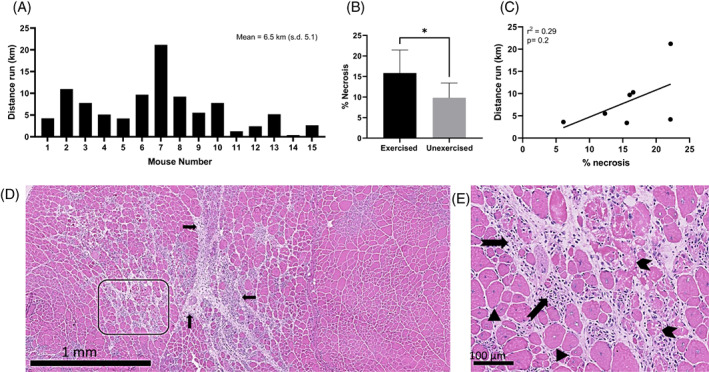
Effect of exercise on *mdx* muscle pathology. A, Distance run by each *mdx* mouse in the study. B, There was a significant difference in the percentage of necrosis between exercised and unexercised mice (*P* = .03). C, Scatterplot shows the relationship between percentage of necrosis and distance run on the running wheel. Although not significant, a clear trend can be observed. D, Low‐magnification image shows regions of necrosis with inflammatory cells (arrows). Relatively spared areas can be seen to the right of the image. E, Higher magnification image of the region shown in C. Small, darkly stained (basophilic) inflammatory cells can be seen (notched arrows), together with hypercontracted and degenerating necrotic myofibers with fragmented sarcoplasm (chevrons). Regenerated fibers with central nuclei can also be observed (arrowheads).

Distances run by the mice were variable, with a group average of 6.5 km (range, 0.4 to 21.2 km; Figure 1). The percentage of necrosis observed was also variable in both the exercised and unexercised mice (exercised range, 15.6% to 22.2%; unexercised range, 4.7% to 13.2%), but voluntary wheel running resulted in a significant increase in muscle fiber necrosis (*P* = .03). There was no significant correlation between muscle necrosis and distance run (*r* ^2^ = .29, *P* = .2; Figure 1C).

**FIGURE 2:**
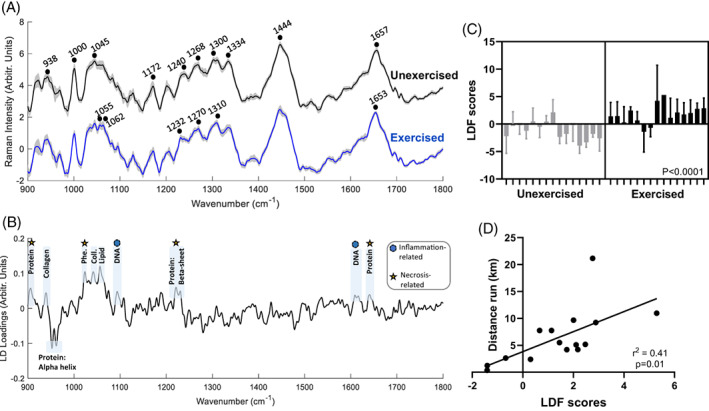
*In vivo* intramuscular Raman spectra in unexercised and exercised mice. A, Average spectra (with standard deviation) of unexercised and exercised *mdx* mice. B, Linear discriminant function (LDF) loading plot for the unexercised vs exercised comparison. Tentative peak and pathology assignments are shown. C, Nested LDF scores for each *mdx* mouse. D, Scatterplot shows the relationship between average LDF score for each *mdx* mouse and distance run on the running wheel.

Raman spectra were composed of peaks relating to α‐helical protein structure (938, 1300, 1654 cm^−1^; Figure 2),^24^, ^25^ phenylalanine (1000 cm^−1^),^26^ and proteins/phospholipids (1444 cm^−1^).^27^ These represent the molecular composition of muscle components such as myosin, tropomyosin, and actin.^5^ Visual inspection of mean spectra revealed differences between exercise and unexercised mice, for example, in peaks relating to protein structure (region 1230 to 1310 cm^−1^)^28^, ^29^ and nucleic acids (1055/1062 cm^−1^).^27^

To identify and quantify exercise‐induced molecular changes in muscle, PCA‐LDA was employed. The shape of the LDF resulting from the PCA‐LDA is shown in Figure 2B. Positive intensities represent peaks more associated with exercised muscle spectra; the most prominent positive peaks were at 904 cm^−1^ (associated with C‐C skeletal stretching with protein^30^), 930 cm^−1^ (associated with collagen^31^), 1025 cm^−1^ (C‐H stretch of phenylalanine^32^), 1045 cm^−1^ (collagen^33^), 1057 cm^−1^ (lipid^34^), 1095 (DNA^35^), 1220 cm^−1^ (protein: β‐sheet^26^), 1610 cm^−1^ (DNA^36^), and 1641 cm^−1^ (protein^27^). Negative intensity peaks represent those more associated with unexercised mice; prominent peaks were seen at 951 cm^−1^ (protein: α‐helix^27^) and 961 cm^−1^ (C‐C stretch in protein^37^). A significant difference in LDF scores was observed between exercised and unexercised spectra (Figure 2C) and LDF scores had a significant correlation with distance run (Figure 2D).

**FIGURE 3:**
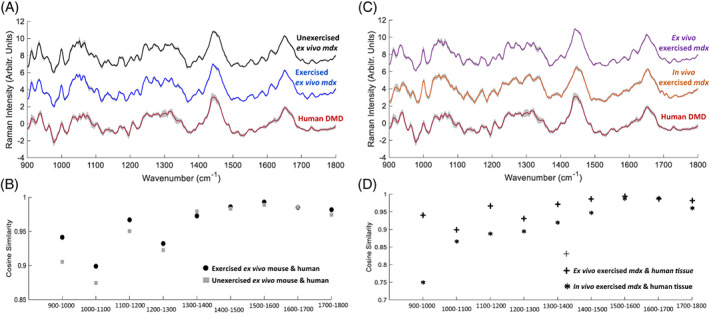
*mdx* and human *ex vivo* Raman spectra. A, Average Raman spectra (with standard deviation) for *ex vivo mdx* gastrocnemius muscle (unexercised and exercised) and human quadriceps muscle. Spectra are vertically offset for clarity. B, Cosine similarity of exercised/unexercised *mdx* spectra and human muscle. The exercised spectra exhibit a slightly higher degree of similarity with human spectra in the lower wavenumber regions. C, Average Raman spectra (with standard deviation) for *ex vivo* and *in vivo mdx* gastrocnemius muscle and human quadriceps muscle. Spectra are vertically offset for clarity. D, Comparison of *ex vivo mdx* and *ex vivo* human spectra (plus sign) consistently demonstrates a greater degree of similarity than the comparison between in vivo *mdx* spectra and ex vivo human spectra (asterisk). This is most apparent in the low‐ to mid‐range wavenumbers (ie, 900 to 1400 cm^−1^).

As the human tissue analysis was performed *ex viv*o, we first undertook a comparison with *ex vivo mdx* gastrocnemius measurements. Greater similarity was observed between exercised *mdx* and human samples than for unexercised *mdx* and human samples (Figure 3). This was most evident in the first half of the spectral window. A further comparison between *in vivo* and *ex vivo* exercised *mdx* spectra and human spectra demonstrated that *ex vivo mdx* spectra more closely resembled the *ex vivo* human tissue (Figure 3D).

## 4 DISCUSSION

We have demonstrated that fiber optic Raman spectroscopy of muscle can quantify the effects of exercise in the *mdx* mouse. We also showed that exercise in this model results in pathology that more closely resembles human disease when analyzed by Raman spectroscopy. Thus, we highlighted the potential of Raman spectroscopy of muscle as a translational tool. The rapid, label‐free nature of Raman spectroscopy, and the portable nature of the fiber optic system, make Raman spectroscopy a promising candidate for further development in neuromuscular disorders.

The distances run by individual *mdx* mice were variable but in keeping with previous studies,^10^, ^11^, ^38^ as was the percentage of necrosis recorded in exercised and unexercised mice^10^ and the general trend of increasing muscle necrosis with running distance.^11^ In *mdx* mice, the response of different muscles to exercise is known to vary, with, for example, quadriceps pathology being exacerbated to a greater degree and tibialis anterior largely unaffected.^18^ We chose to focus our analysis on gastrocnemius as *in vivo* fiber optic Raman measurements can be easily performed on this muscle and it is known to respond to exercise.^10^ With some minor modifications to the recording protocol, other muscles, such as quadriceps, may be amenable to *in vivo* recordings, thus expanding the potential of the technique.

The Raman spectra appear to contain peaks relevant to the underlying histopathology.^39^ Although necrosis has not yet been studied in muscle using Raman spectroscopy, it has been examined in other models/organs. For example, necrosis in brain and cell culture systems has been reported to result in an increase in protein‐related peaks.^40^ In our analysis, peaks suggesting structural modifications to proteins were seen, with β‐sheet–related peaks observed in the exercised group. Muscle contains a large number of proteins in which the α‐helix confirmation is the predominant secondary protein structure.^41^ During protein deformation, a transition to β‐sheet structures is a common occurrence^42^ and has been reported previously in dystrophin‐related muscle pathology.^43^ In our experimental paradigm, we postulate that it may be related to increased myofiber breakdown that may either precede or occur in association with necrosis. In addition, in the average spectra plots, the amide I protein peak was seen at 1657 cm^−1^ in unexercised mice and 1653 cm^−1^ in exercised mice. This also suggests a structural modification of protein, which, interestingly, has previously been reported in Raman studies on necrosis.^44^ Some nucleotide‐related peaks were also evident in the exercised group, possibly related to the hypercellularity associated with the inflammatory response.^45^ A subset of lipids may have also demonstrated an increase (peak 1057 cm^−1^); it is unclear whether this relates to more cell membranes, or alterations in lipid metabolism.

The greater statistical significance between the exercised and unexercised groups and relationship to running distance seen with Raman, when compared with histological analysis, is not unexpected. Raman spectroscopy collects a biochemical fingerprint based on the molecular composition of the tissue, thus obtaining information from many different molecules. The application of multivariate statistics then enables quantification across diverse molecular features. This is more akin to, for example, wide‐ranging proteome analyses, which in *mdx* studies find greater differences between exercised/unexercised mice than basic histology alone.^46^

Short bursts of high‐intensity exercise in *mdx* are sometimes undertaken to make the muscle pathology more closely resemble the human disease^11^, ^47^, ^48^, ^49^ and, while the effects of exercise in the *mdx* model are complex,^50^ it is encouraging that exercise increased the similarity between *mdx* and human Raman spectra. When comparing mouse and human spectra, it is notable that the greatest increases in similarity were seen in the first half of the spectral window (ie, 900 to 1300 cm^−1^), which also contained the more prominent peaks in the LDF (Figure 3). That *ex vivo mdx* Raman spectra were more similar to human muscle than *in vivo mdx* Raman spectra is to be expected, as both *ex vivo mdx* muscles and human samples were snap frozen and then thawed, a process that can cause some subtle alterations to Raman spectra.^51^

There are several limitations to this proof‐of‐concept study. Voluntary wheel running is simple to implement but results in inter‐mouse variation in the amount of exercise performed. Other exercise paradigms, such as treadmill running,^47^ may lead to a more consistent effect and thus be easier to detect with both Raman spectroscopy (and traditional histology). In some biomedical applications of Raman spectroscopy, the analysis is targeted to areas of interest, identified, for example, by prior histological analysis.^52^ This was not the case in our study. As a result, we may have inadvertently missed areas of interest (eg, necrosis) in both exercised and unexercised mice, potentially resulting in either an underestimation or overestimation of the ability of the Raman method to detect pathology. By collecting four spectra from each mouse (two from each gastrocnemius), the chances of missing/detecting relevant pathology should hopefully even out between the groups. Although gastrocnemius muscles have been shown to manifest exercised‐induced pathology, quadriceps muscles are reportedly more susceptible to exercise effects.^18^ Thus, other muscles could be considered for future analyses. In the present study we attempted recording from heart muscle but found that the background fluorescence was such that no biological information could be seen (not shown). However, Raman spectroscopy has been used to study cardiac tissue,^53^ so alterations to the recording equipment (eg, different laser excitation wavelengths) may be worth pursuing for the study of DMD‐related changes in cardiac muscle.

In this work we have utilized spontaneous Raman spectroscopy, which is arguably the simplest variant of Raman spectroscopy to implement. However, the signal arising from spontaneous Raman is weak, as only around 1 per 10^6^ to 10^10^ photons are inelastically scattered. Increasing the laser power or recording time would increase the intensity of the signal obtained (by collecting more Raman scattered light) but would also increase the signal background. Such changes may be worth evaluating in future studies. It is also possible that, independent of signal/noise effects, extremely subtle chemical changes may still elude a spontaneous Raman approach by being below the limit of detection. Thus, other Raman techniques may be worth evaluating for the detection and quantification of muscle pathology. Surface‐enhanced Raman significantly boosts the signal but would require injection of nanoparticles into the tissue of interest.^54^ Coherent anti‐Stokes Raman spectroscopy uses two beams of light to excite the sample and has been shown to effectively map out protein distributions in human muscle biopsy samples from a range of diseases.^55^

Notwithstanding the potential difficulties with using spontaneous Raman, it appears our present approach can provide an *in vivo* quantification of muscle pathology in preclinical models such as *mdx*. Currently used *in vivo* measures of disease include wire‐hang and grip strength, which can be confounded by animal weight and behavior.^9^ Outputs concerning the structure of muscle, such as magnetic resonance imaging (MRI)^56^ and electrical impedance myography (EIM),^57^ avoid such factors but require expensive, specialized equipment (ie, MRI), or do not directly assess biochemical/molecular changes (ie, EIM). Thus, many studies rely on *ex vivo* muscle force and histological studies. The spontaneous fiber optic Raman method also has the potential to translate to *in vivo* human recordings with relatively few modifications to the present equipment.

In conclusion, our results indicate that fiber optic Raman spectroscopy is a promising tool for the quantification of muscle pathology in DMD. The technique could provide an *in vivo* readout of disease state in preclinical models, and it may be possible in the future to undertake such recordings in human patients.

## FUNDING INFORMATION

Academy of Medical Sciences (starter grant to J.J.P.A.); Medical Research Council (Confidence in Concept Awards to J.J.P.A., J.C.D., R.J.M., P.J.S.); National Institute for Health Research (NIHR) (Senior Investigator Award to P.J.S.); NIHR Sheffield Biomedical Research Centre (to P.J.S.).

## CONFLICT OF INTEREST

The authors report no conflicts of interest.

## ETHICAL PUBLICATION STATEMENT

The authors confirm that they have read the Journal's position on issues involved in ethical publication and affirm that this report is consistent with those guidelines.

## Supporting information


**APPENDIX S1** Supplementary figures

## Data Availability

The data that support the findings of this study are available from the corresponding author upon reasonable request.
