# Supplementary material for: Fiber optic Raman spectroscopy for the evaluation of disease state in Duchenne muscular dystrophy: An assessment using the mdx model and human muscle
Source: Muscle Nerve. 2022 Jul 15;66(3):362–9. doi: 10.1002/mus.27671 (PMC9541045; doi:10.1002/mus.27671)
Supplement: Supplementary file 1 — APPENDIX S1 Supplementary figures [file MUS-66-362-s001.zip › 3rd Revision_Supple fig legends_CLEAN.docx]

**Supplemental figure 1.**

Raman spectroscopy overview.

Light of a single wavelength interacts with molecules in the sample. A small proportion of photons (around 1 in 10^6^-10^10^) imparts or absorbs energy from vibrational states (the periodic motion of atoms relative to each other) in the molecules. Since a photon’s energy is a function of wavelength, this causes a shift in the wavelength of the photon, represented by coloured arrows with dot/dash lines. This is termed inelastic or Raman scattering. This differs from more abundant elastic scattering (Rayleigh scattering), in which the energy of the light is not changed (complete, black arrows). Note: molecules are for illustration only, they do not represent specific molecular configurations.

Raman scattered light is collected and a spectrum plotted. Biological Raman spectra are complex, specific peaks are assigned to specific molecular bonds and different biological molecules. When comparing health and disease new peaks may appear, they may shift along the x-axis, or change shape.

Thus, a Raman spectrum contains detailed information which can be conceptualised as a molecular fingerprint of the sample. As changes in disease can be subtle, advanced statistical techniques (‘chemometrics)’ are applied to understand the information contained within the spectra.

Interested readers are directed to comprehensive reviews^1-4^.

**Supplemental figure 2.**

Fibre optic Raman spectroscopy equipment.

A fibre optic coupled laser provides the incident light which enters a bandpass filter, this removes any Raman scattered light and/or fluorescence generated in the initial part of the optical pathway. The light is then focused into an excitation fibre housed within a standard hypodermic needle. An identical second fibre acts as the collecting fibre; both elastically and inelastically scattered photons enter this fibre and pass to a filter that rejects the elastically scattered light. A fibre optic coupled spectrometer and charged coupled device (CCD) camera detect the inelastically scattered light and record the Raman spectrum.

**Supplemental figure 3.**

Histological image of unexercised mdx muscle. Note that the same pathology appears in exercised (see figure 2) and unexercised mice e.g. centrally nucleated regenerating fibres and necrotic fibres.

**Supplemental references**

1. Hanlon EB, Manoharan R, Koo TW, Shafer KE, Motz JT, Fitzmaurice M, Kramer JR, Itzkan I, Dasari RR, Feld MS. Prospects for in vivo Raman spectroscopy. Phys Med Biol 2000;45(2):R1-59.

2. Butler HJ, Ashton L, Bird B, Cinque G, Curtis K, Dorney J, Esmonde-White K, Fullwood NJ, Gardner B, Martin-Hirsch PL et al. Using Raman spectroscopy to characterize biological materials. Nat Protoc 2016;11(4):664-687.

3. Morais CLM, Lima KMG, Singh M, Martin FL. Tutorial: multivariate classification for vibrational spectroscopy in biological samples. Nat Protoc 2020;15(7):2143-2162.

4. Guo S, Popp J, Bocklitz T. Chemometric analysis in Raman spectroscopy from experimental design to machine learning-based modeling. Nat Protoc 2021;16(12):5426-5459.
