# Supplementary figures and images for: Fiber optic Raman spectroscopy for the evaluation of disease state in Duchenne muscular dystrophy: An assessment using the mdx model and human muscle
Source: Muscle Nerve. 2022 Jul 15;66(3):362–9. doi: 10.1002/mus.27671 (PMC9541045; doi:10.1002/mus.27671)

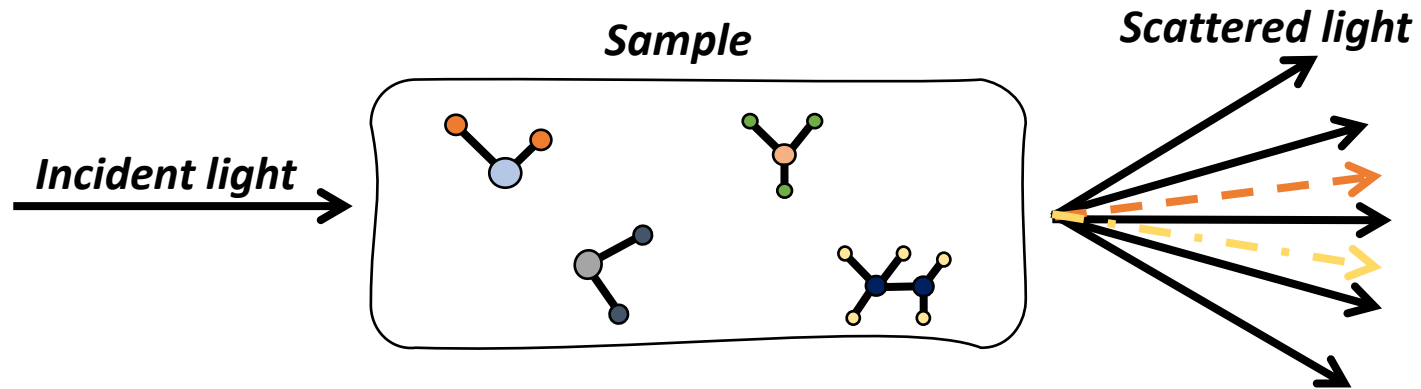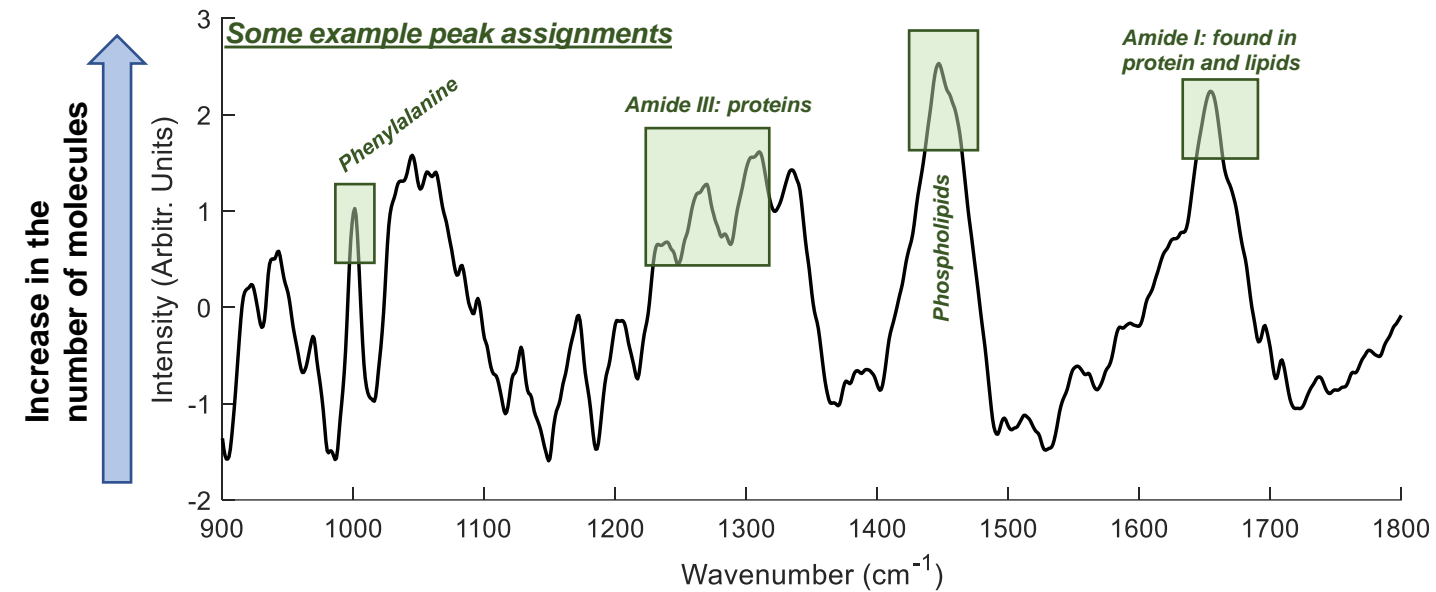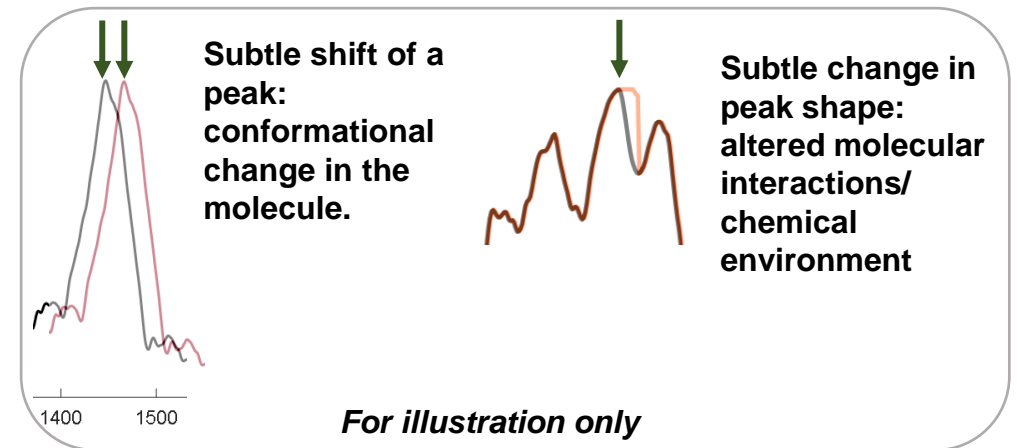

Supplement: Supplementary file 1 — APPENDIX S1 Supplementary figures [file MUS-66-362-s001.zip › Supplementary figure 1.pdf]

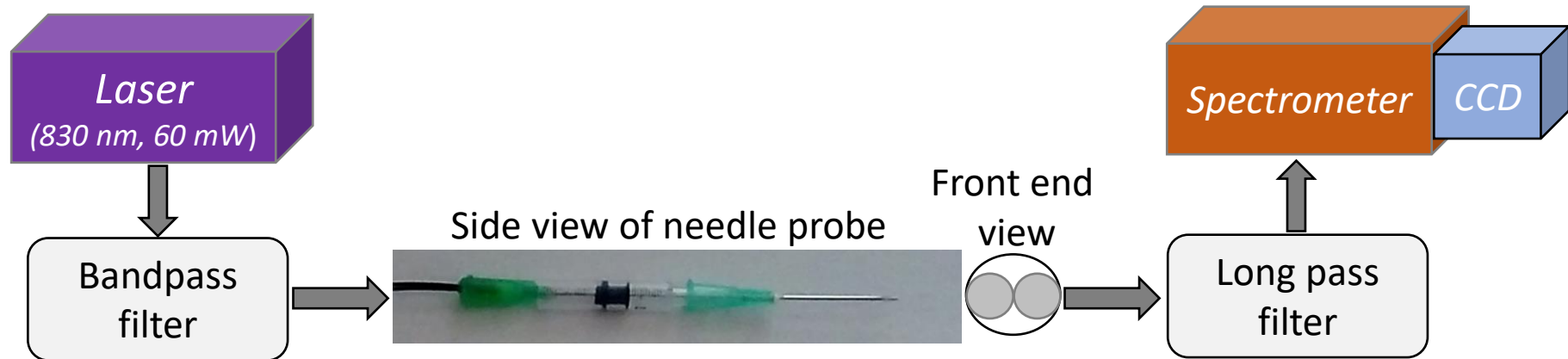

Supplement: Supplementary file 1 — APPENDIX S1 Supplementary figures [file MUS-66-362-s001.zip › Supplementary figure 2.pdf]

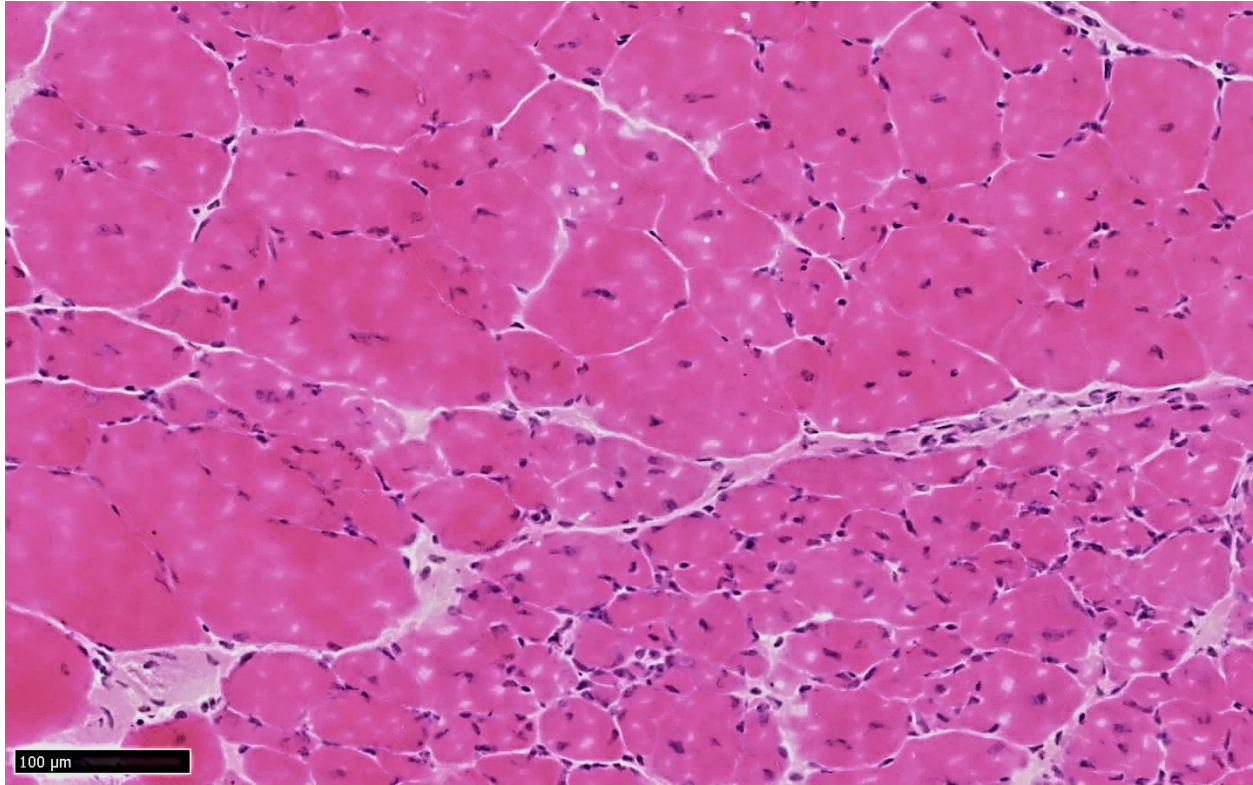

Supplement: Supplementary file 1 — APPENDIX S1 Supplementary figures [file MUS-66-362-s001.zip › Supplementary figure 3.pdf]
